# The Significance of Redox Biomarkers in the Evaluation of the Antioxidant Profile In Vitro and In Vivo

**DOI:** 10.3390/antiox10050805

**Published:** 2021-05-19

**Authors:** Aristidis S. Veskoukis, Periklis Vardakas, Dimitrios Kouretas

**Affiliations:** 1 Department of Nutrition and Dietetics, School of Physical Education, Sport Science and Dietetics, University of Thessaly, Argonafton 1, 42132 Trikala, Greece; veskoukis@uth.gr; 2 Department of Biochemistry and Biotechnology, University of Thessaly, Viopolis, Mezourlo, 41500 Larissa, Greece; periklis_vardakas94@hotmail.com

The present Special Issue of *Antioxidants*, entitled *The Significance of Redox Biomarkers in the Evaluation of the Antioxidant Profile In Vitro and In Vivo*, ten research articles emphasize the significance of adopting reliable redox biomarkers to determine the antioxidant activities of bioactive compounds in vitro and to assess blood and tissue redox status in vivo. First, Chaouni et al. [1] and de Toledo et al. [2] highlight the ability of redox biomarkers to depict the potential modifications of redox homeostasis due to therapeutic chemical and nutritional interventions in vivo. Then, four articles (i.e., two papers from Toczewska et al. [3,4], Maciejczyk et al. [5] and Bourgonje et al. [6]) describe the role of specific redox biomarkers as clinical outcomes of diseases and, concomitantly, examine their putative value in disease diagnosis. Furthermore, Bouroutzika et al. [7], Matute et al. [8] and Islam et al. [9] demonstrate the beneficial properties of endogenous antioxidant compounds and phytochemicals on physiological processes, as well as their ability to protect from toxic agents and stressors, associated with oxidative stress induction. Finally, Karydas et al. [10] describe the development of a novel multi-parametric system for the effective assessment of fruit antioxidant content, giving clues regarding precision farming. 

The use of proton beams has been recently introduced as an alternative strategy to conventional radiotherapy against cancer and, to develop the research on this strategy, Chaouni et al. [1] have highlighted the necessity of investigating its potential adverse effects on healthy tissues. In this context, the biological role of two main techniques of proton irradiation were assessed based on biomarkers of inflammation, oxidative stress and genotoxicity, using a murine model. According to the results, proton irradiation induced genotoxic effects and perturbations on the redox state of blood and tissues which, of note, persisted well after exposure. Furthermore, de Toledo et al. [2] have demonstrated the beneficial properties of fasting, a modern and attractive nutritional mode, on humans. In particular, the authors have pointed out that a 10-day fasting program not only improved numerous crucial clinical parameters, but also improved the blood redox profile of the participants, hence promoting metabolic health. This study sets the foundation for the need to further evaluate the clinical and redox-related positive effects of long-term fasting. 

It is a fact that the pathophysiology of various diseases is redox-related, given that the impairment of redox equilibrium of blood and tissues is a clinical outcome. Therefore, the development of reliable redox biomarkers towards early diagnosis and evaluation of disease progression is a major challenge for the researchers in the field of Redox Biology. Approaching this essential issue, Toczewska et al. [3] have attempted to correlate salivary redox biomarkers with the advancement of periodontitis, an oral disease that has been characterized by chronic inflammatory responses and disturbance of redox equilibrium. Indeed, the results of this study have pinpointed elevated levels of nitrosative stress biomarkers in the saliva of periodontitis patients. In their second relevant study, Toczewska et al. [4] have investigated the linkage between saliva redox biomarkers and gingival crevicular fluid and periodontitis. They concluded, however, that the studied biomarkers did not correlate with clinical periodontal status. Additionally, Maciejczyk et al. [5] have endeavored to develop a reliable redox biomarker, adequately reflecting the stage of progression of chronic kidney disease, which is another pathological condition associated with perturbations of redox homeostasis. The authors have demonstrated a differentiation of salivary total antioxidant potential not only between healthy individuals and patients, but also among patients with different levels of disease severity, indicating the possibility that, under specific circumstances, it could be considered as a putative diagnostic tool. Finally, Bourgonje et al. [6] have evaluated the accuracy of serum free thiols for monitoring patients with inflammatory bowel diseases, whose etiology is also linked to oxidative stress, instead of using conventional fecal biomarkers. It has been concluded that the determination of the levels of serum free thiols could be regarded as potential non-invasive strategy with respect to disease monitoring, since their levels differ significantly between healthy subjects and patients, and they are also indicative of the disease severity. 

The negative effects of oxidative stress can be counterbalanced by antioxidants, hence the supplementation of bioactive compounds with antioxidant properties has risen to the forefront of scientific interest during the last decades. In this regard, Bouroutzika et al. [7] have investigated the ability of melatonin to ameliorate the effects of heat stress, which has been associated with induction of detrimental oxidative modifications in biomolecules, during the pregnancy period of ewes. According to the findings of this study, the administration of melatonin throughout gestation enhanced the redox antioxidant resources available to the animals, while the parameters associated with reproduction were also improved. These results imply an important positive correlation between biomarkers of antioxidant status and the reproduction process of productive animals. Matute et al. [8] have examined the impact of a multitude of polyphenol-rich fruit and vegetable juices on the vascular activity of an ex vivo model in terms of their phenolic content. Towards this direction, they stressed that three phenolic compounds, specifically kaempferol, epigallocatechin gallate and peonidin-3-*O*-glucoside, appear to be probably the main contributors of the vasorelaxing action observed during fruit juice consumption. Additionally, Islam et al. [9] have appraised the protective properties of a traditional Chinese medicinal plant, namely *Illicium henryi*, against the lipopolysaccharide-induced acute liver damage in a murine model. The results of their study suggested that the therapeutic efficacy of the corresponding plant extract, which also exerted in vitro antioxidant activities, was attributed to the upregulation of Nrf2 and the downregulation of TLR4/NF-κB signaling pathways, hence providing hepatoprotection against oxidative damage and inflammation. 

Finally, Karydas et al. [10] have developed a novel machine-learning model able to predict the antioxidant potential of cherry fruits, using a set of data acquired from two years of cultivation. Particularly, the model development was achieved via analysis of the antioxidant properties of the fruits along with the use of multispectral images captured from drones, soil analysis data, topographic, hydrographic and climatological data. The obtained results showed that soil parameters and weather data are key factors regarding the antioxidant properties and the total phenolic content of the fruits. It is concluded that this newly developed system can be utilized to support site-specific treatments (precision farming) for improving fruit quality in the long-term, with noteworthy marketing perspectives.

Τhe studies included in this Special Issue cover a wide range of topics that converge in highlighting the significance of adopting redox biomarkers in order to approach modern research issues both in vitro and in vivo. Undisputedly, redox biomarkers comprise a powerful tool to monitor the efficacy of antioxidant molecules in vitro and to evaluate the modifications of redox status in health and disease in in vivo models. Thus, the measurement of specific redox biomarkers is directed to shed light on complex queries in the field of Redox Biology. 
